# In vitro and in vivo dissolution of biocompatible S59 glass scaffolds

**DOI:** 10.1007/s10856-024-06795-x

**Published:** 2024-07-03

**Authors:** Laura Aalto-Setälä, Peter Uppstu, Robert Björkenheim, Gustav Strömberg, Nina C. Lindfors, Jukka Pajarinen, Leena Hupa

**Affiliations:** 1 Johan Gadolin Process Chemistry Centre, Åbo Akademi University, Turku, Finland; 2 Polymer Technology Research Group, Faculty of Science and Engineering, Åbo Akademi University, Turku, Finland; 3 Department of Musculoskeletal and Plastic Surgery, Helsinki University Hospital, Helsinki University, Helsinki, Finland; 4 Helsinki University, Helsinki, Finland

## Abstract

Fabrication of porous tissue-engineering scaffolds from bioactive glasses (BAG) is complicated by the tendency of BAG compositions to crystallize in thermal treatments during scaffold manufacture. Here, experimental biocompatible glass S59 (SiO_2_ 59.7 wt%, Na_2_O 25.5 wt%, CaO 11.0 wt%, P_2_O_5_ 2.5 wt%, B_2_O_3_ 1.3 wt%), known to be resistant to crystallization, was used in sintering of glass granules (300–500 µm) into porous scaffolds. The dissolution behavior of the scaffolds was then studied in vivo in rabbit femurs and under continuous flow conditions in vitro (14 days in vitro/56 days in vivo). The scaffolds were osteoconductive in vivo, as bone could grow into the scaffold structure. Still, the scaffolds could not induce sufficiently rapid bone ingrowth to replace the strength lost due to dissolution. The scaffolds lost their structure and strength as the scaffold necks dissolved. In vitro, S59 dissolved congruently throughout the 14-day experiments, resulting in only a slight reaction layer formation. Manufacturing BAG scaffolds from S59 that retain their amorphous structure was thus possible. The relatively rapid and stable dissolution of the scaffold implies that the glass S59 may have the potential to be used in composite implants providing initial strength and stable, predictable release of ions over longer exposure times.

**Graphical Abstract:**

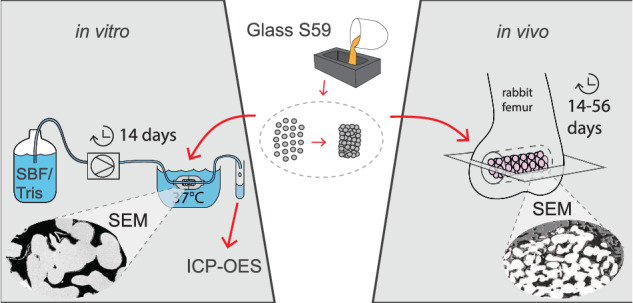

## Introduction

Bioactive glasses have widely been studied for bone grafting and healing applications since Hench et al. reported the concept in 1971 [1]. Since then, many different bioactive glass compositions and applications have been developed [2–8]. Different silicate-based bioactive glass compositions share the same stages of surface reactions in aqueous solutions, namely selective leaching of alkali ions from the glass surface, followed by glass-network dissolution, condensation, and repolymerization of a silica-rich layer until finally, a calcium phosphate layer precipitates at the glass surface [2]. In addition to being osteoconductive, the Si, Ca, and P ion species released from the dissolving BAG support osteogenesis and angiogenesis [9, 10]. BAG is traditionally applied as granules or paste as bone grafting materials (would this be better?). Recently, there has been growing interest in fabricating porous tissue-engineering scaffolds from bioactive glasses [11–18] (for thorough reviews, see e.g., [19, 20]). The main challenge is that many silica-based bioactive compositions, such as the clinically used Bioglass® 45S5 and BonAlive® S53P4, crystallize easily in thermal treatments typically needed during scaffold manufacture [21, 22]. As crystallization changes bioactivity [23], many efforts have been directed toward developing BAG compositions and sintering methods with lower crystallization tendency [17, 24–28]. Recently, there has been significant interest in substituting therapeutic inorganic ions, such as zinc [29, 30], strontium [31, 32], magnesium [33, 34], [35], copper [36], or boron [5, 37] for the ions in known bioactive glasses. These additional ions dissolving from bioactive glasses are known to possess various positive effects in new bone formation and angiogenesis for implanted glasses [38, 39].

The advantage of a more slowly dissolving glass is to release therapeutic ions over a more extended period, stimulating the targeted cellular processes for a longer time [25]. Less-reactive bioactive glasses also provide a steadier initial ion release than more bioactive compositions under in vitro conditions [40], which implies that even the initial release dosage remains controllable.

The more slowly dissolving glass compositions have a higher concentration of glass formers than the more reactive bioactive glasses, making them less sensitive to crystallization during sintering; they can be sintered to a higher degree without crystallization than the more reactive compositions [41]. These biocompatible melt-derived glasses can be produced into porous, strong, and amorphous scaffolds.

This study evaluated the suitability of an experimental, biocompatible glass S59 composition for porous scaffold manufacture and bone repair applications. The composition is biocompatible in vivo in granule form [42] and has a low crystallization tendency [43]. The composition was chosen for its established biocompatibility, crystallization resistance, and low reactivity compared to some better-known bioactive glasses with low crystallization tendency (i.e., 13–93). The lower reactivity was desirable to have a more controlled and prolonged degradation of the scaffolds.

We report both in vivo and in vitro reactions of sintered S59 scaffolds. The overall dissolution rate of the glass was studied in vitro using a continuous flow-through system to provide further insight into the degradation behavior of the composition. The continuous flow in vitro study was chosen over static tests to mimic the in vivo conditions better [43] and to avoid SBF-related challenges, such as excessively rapid layer formation, present in static immersion [45–48]. In the in vivo study, the main goal was to explore bone ingrowth into the porous biocompatible scaffolds. The results give insights into the role of slowly dissolving glass scaffolds in providing temporary initial support and guiding tissue ingrowth.

## Materials and methods

### Scaffold preparation and characterization

Glass S59 (nominal composition in wt% SiO_2_ 59.7, Na_2_O 25.5, CaO 11.0, P_2_O_5_ 2.5, B_2_O_3_ 1.3) was melted at 1360 °C in a platinum crucible for 3 h from analytical grade reagents (Na_2_CO_3_, CaCO_3_, 2H_2_OCaHPO_4_, H_3_BO_3_) and Belgian glass quality quartz sand. Glass was melted twice to ensure homogeneity and cast into a block that was annealed overnight. The annealed glass block was then crushed and sieved to yield granules of size fraction 300–500 μm. The granules were sintered in a graphite mold into cylinders (for the in vivo study, height 15 mm, diameter 5 mm; for the in vitro, height 10 mm, diameter 5 mm) in a nitrogen atmosphere for 90 min at 630 °C.

The amorphous nature of the sintered scaffolds was verified using Empyrean X-ray diffractometer (Malvern Panalytical, Almelo, The Netherlands, Cu α radiation, 40 mA, 40 kV, 10–80° 2Ɵ, 2.0°/min). The scaffolds were powdered with an agate mortar and pestle before the analysis.

The porosity was determined from five cross-sectional SEM images of the scaffolds at ×30 magnification using Photoshop CS6 software (Adobe Systems Inc, San Jose, CA, USA).

### In vitro dissolution tests

**Fig. 1:**
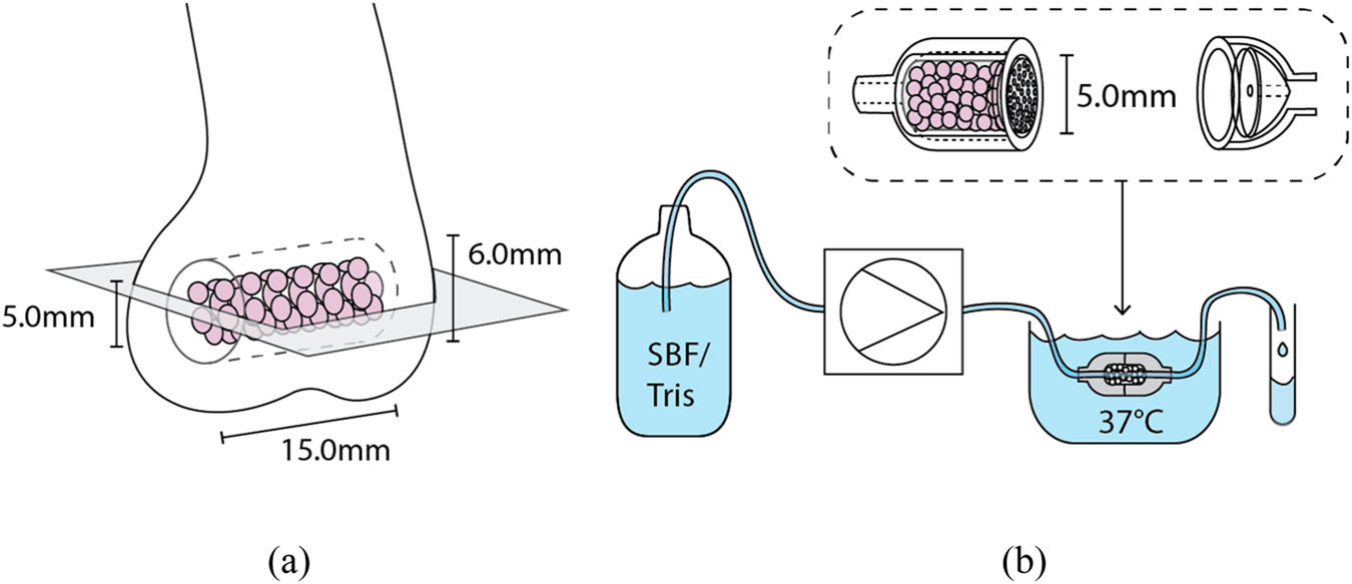
Schematic images showing (**a**) the in vivo implantation site in a rabbit femur and the imaged cross-sectional plane (gray), and (**b**) the setup for the continuous in vitro study. Images not to scale

The dissolution of S59 granules and sintered scaffolds was studied in vitro under dynamic immersion in a reactor setup, through which a fresh buffer solution was continuously fed at the average rate of 0.2 ml/min. For each sample type, there were two test runs. Fagerlund et al. [4] described the reactor cell configuration in detail, and the schematics are shown in Fig. 1b. The flow rate was assumed to mimic a typical laminar flow of the extracellular fluid rate in the human body fluids [4]. Simulated body fluid (SBF) and Tris buffer (pH 7.40) at 37 °C were used as the test solutions. SBF was prepared using the protocol developed by Kokubo et al. [5]. The pH of the Tris buffer solution (50 mM, Trizma base, Sigma-Aldrich) was adjusted with 1 M HCl (J.T. Baker).

The sample mass varied from 264 to 285 mg. The solution feed through the glass sample was continued for up to 14 days. The samples were kept at 37 °C throughout the experiment. The total amount of the solution fed through the glass sample was measured, and the concentrations of the inorganic ions in the solution were analyzed from short-interval (15 min) solution samples collected throughout the experiment at intervals of 1 to 3 days up to 14 days. At each time point, three subsequent samples were collected. These short-interval samples provide momentary concentrations of the ions present in the solution and, together with the total solution volume, enable an estimation of the total dissolution of the glass during the test. The pH was measured from all solution samples.

Ion concentrations of the short-interval samples were analyzed using an inductively coupled plasma optical emission spectrometer (ICP-OES, Optima 5300 DV; Perkin Elmer, Waltham, MA). The samples were diluted at a ratio of 1:9 using ultrapure water. The elements analyzed were silicon (*λ* = 251.611 nm), calcium (*λ* = 317.933 nm), and sodium (*λ* = 589.592 nm). The glass also contained phosphorus and boron, but the observed concentrations were too low to yield reliable data after the first days of dissolution. Thus, these elements were omitted from the analyses. The calibration was conducted using ultrapure water and multielement standards (Perkin Elmer Multielement Standard 25 and silicon standard from Ultra Scientific) with 1 ppm concentrations of Si, Ca, Na, and K. The calibration was rechecked after every 20 samples. All reported values are background corrected. The ion release was assumed to be linear between each time point measured when estimating the cumulative dissolution.

### In vivo study

Our previous study described the surgical procedure in more detail [49] (Animal Experimental Board of Finland permit number ESAVI/440/04.10.07/2014). In short, the scaffolds were sterilized by gamma irradiation (dose 25 kGy) before implantation into 6.0-mm diameter holes drilled into the metaphysis of rabbit femurs for 14, 28, or 56 days (schematic image shown in Fig. 1a). There were three rabbits at each time point (i.e., nine rabbits in total). After the in vivo experiment, the parts of the femurs with implanted scaffolds were cut, dried, and cast into epoxy resin.

### Implant characterization

The femurs with scaffolds were ground and polished using SiC papers after implantation. The longitudinal cross-sections of the polished samples were analyzed with a Leo Gemini 1530 SEM instrument (Carl Zeiss, Oberkochen, Germany). The cross-sectional plane is shown in a schematic image in Fig. 1a. Panorama images of the scaffolds were taken at ×75 magnification. After immersion, the in vitro granules and scaffolds were treated using the same post-processing procedure. Three scaffolds were cast into epoxy resin directly after sintering for zero-day references.

The different reaction layers of the S59 scaffolds after in vivo implantation (i.e., unreacted glass, silica-rich layer, hydroxyapatite (HA) layer, and bone tissue) were identified using EDX analysis. Different tissue types were visually separated from the SEM panorama images and marked with different colors using Photoshop CS6 software (Adobe Systems Inc, San Jose, CA, USA). The amounts of pixels belonging to each tissue type were calculated using a script written in GNU Octave software.

## Results

### Scaffold characterization

**Fig. 2:**
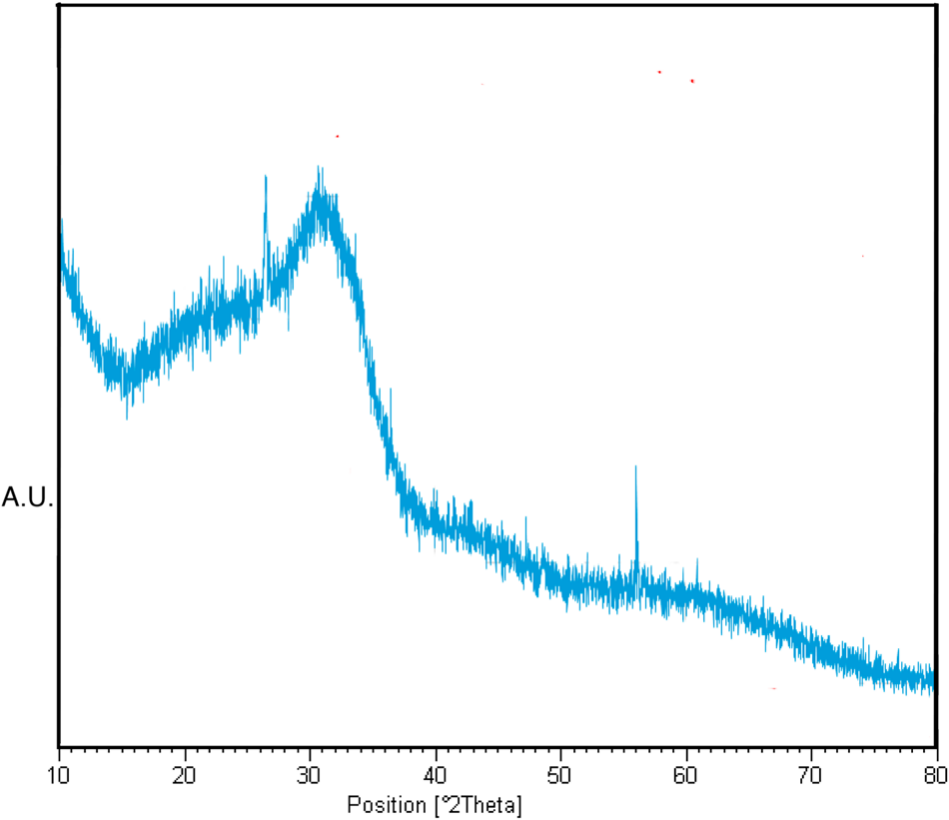
XRD analysis for sintered S59 scaffolds

Figure 2 shows the diffractogram of the scaffolds sintered at 630 °C for 90 min. No distinct signs of crystalline phases were detected apart from the peaks at 26.5 and 54.5° characteristic of graphite, likely due to contamination from the graphite mold [50]. The compressive strength of the scaffolds was 11.1 MPa [43]. The porosity of the scaffolds was calculated to be 32.6 ± 3.2%.

### In vitro reactions

**Fig. 3:**
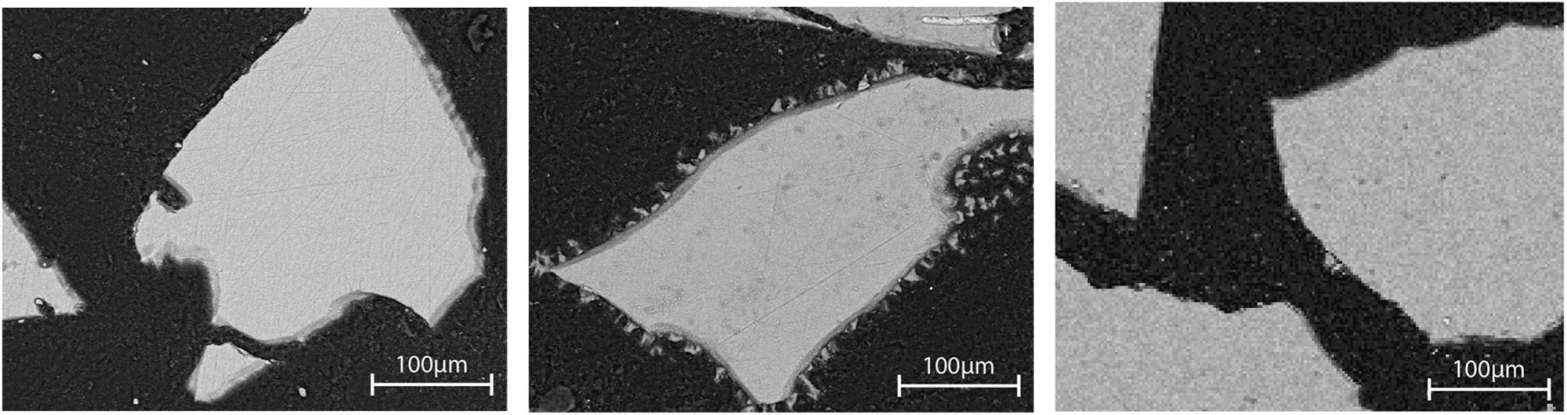
Glass S59 granules after (**a**) 3 days (left) and (**b**) 7 days in continuous flow (0.2 ml/min) of SBF (middle), and (**c**) after 14 days in continuous flow (0.2 ml/min) of Tris (right)

**Fig. 4:**
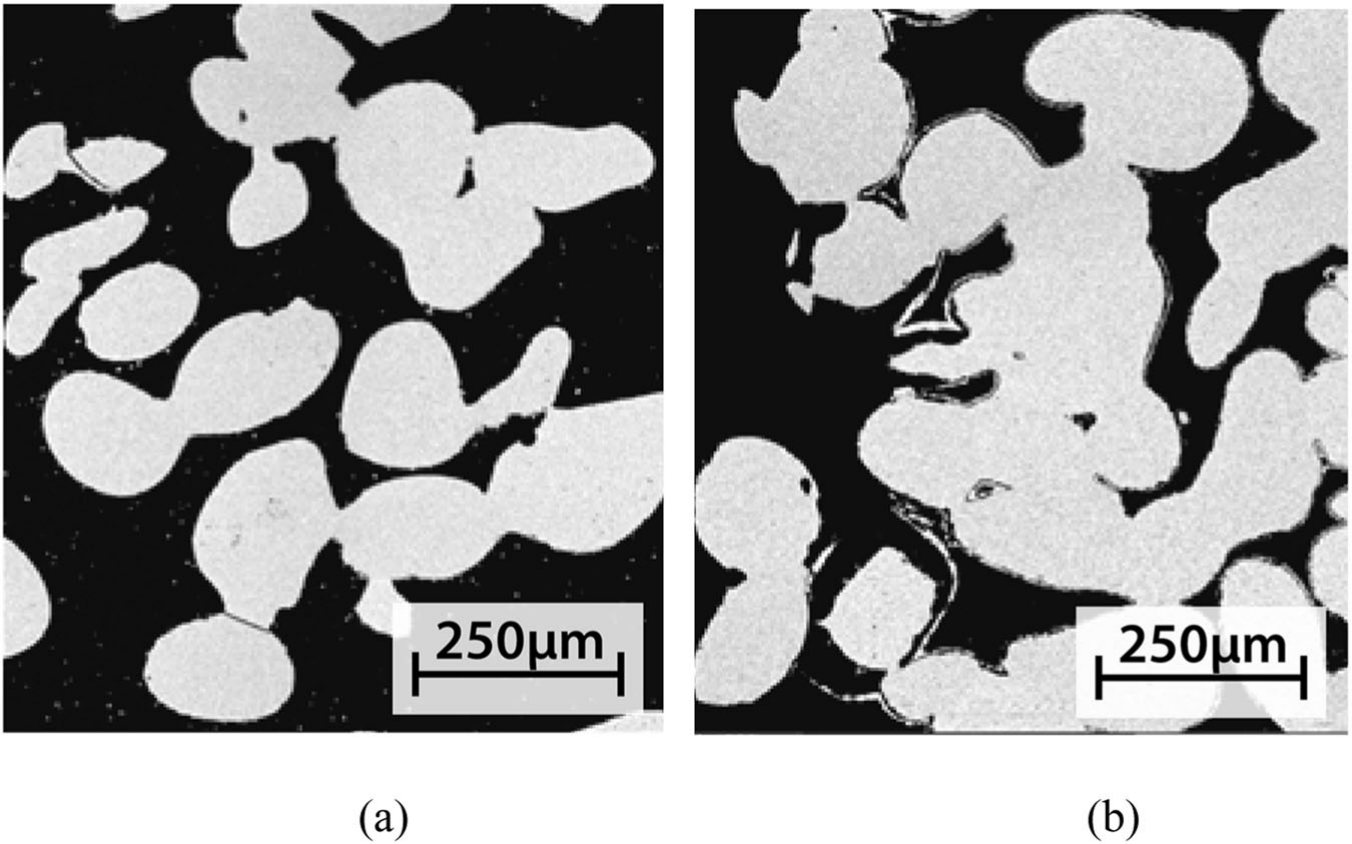
SEM images of S59 scaffold cross-sections after 14 days in (**a**) Tris and (**b**) SBF

Glass S59 granules and scaffolds showed only minor surface layer formation during testing in the continuous flow of the two buffered solutions (Fig. 3 for S59 granules and Fig. 4 for S59 scaffolds).

For granules in SBF, a thin Si-rich layer was seen after 3 days; indications of a slight calcium phosphate (CaP) precipitation were shown after 7 days (Fig. 3a, b). For scaffolds in SBF, there was only a thin CaP layer around the outermost scaffold granules after 14 days in SBF (Fig. 4b).

For granules in Tris, there was an almost negligible Si-rich layer and no indication of a CaP layer formation even after 14 days (Fig. 3c). Similarly, for scaffolds in Tris, no reaction layers could be identified in the SEM images (Fig. 4a).

**Fig. 5:**
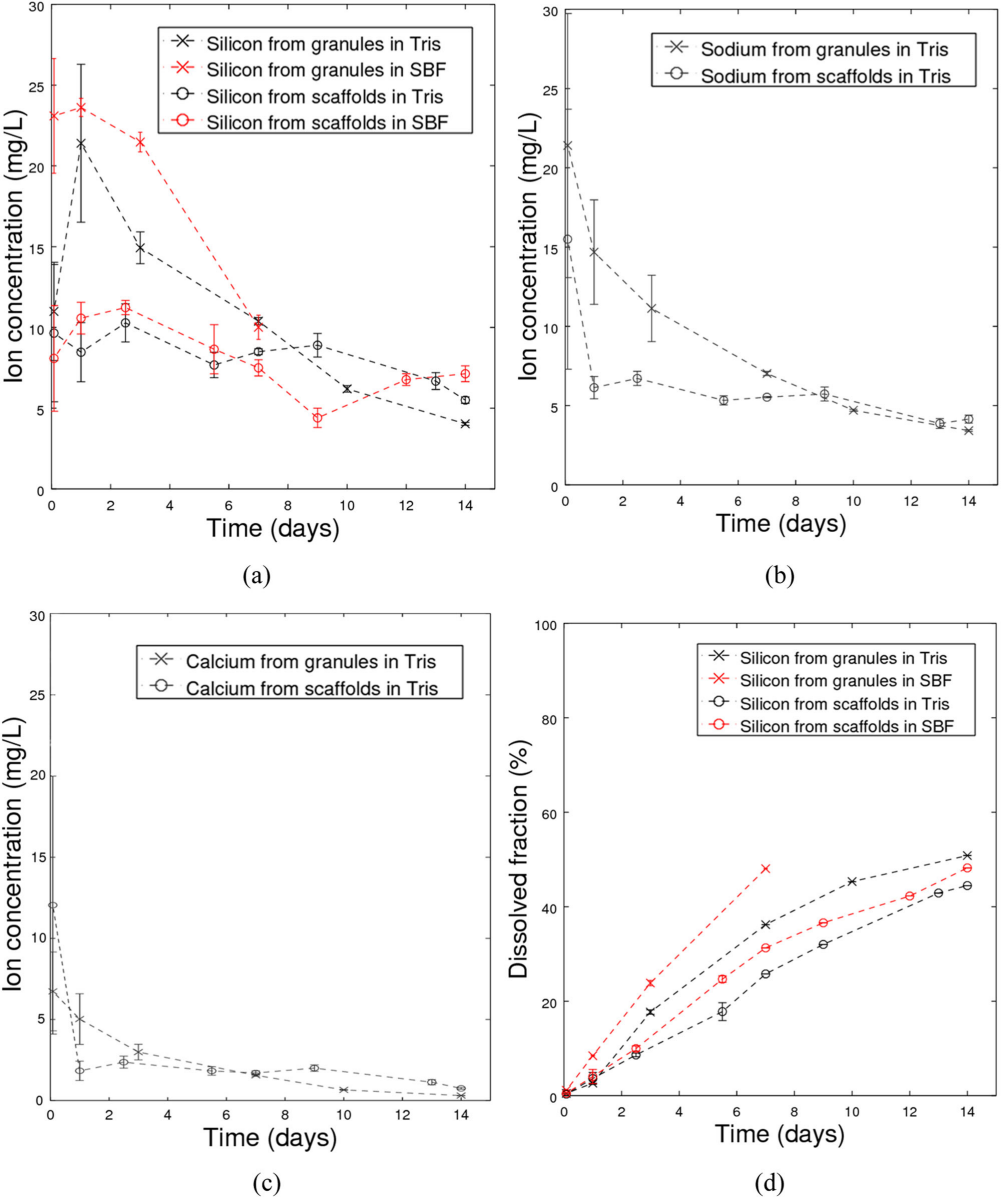
Concentrations of (**a**) silicon, (**b**) sodium, and (**c**) calcium released from glass S59 as functions of time in continuous flows (0.2 ml/min) of Tris and SBF, and (**d**) cumulative silicon dissolution of glass S59 in the solutions. The concentrations are momentary, not cumulative, i.e., those analyzed in the solution outflow at the measuring timepoint

The ICP results (shown for silicon Fig. 5a, sodium Fig. 5b, and calcium Fig. 5c) showed that the granules and scaffolds dissolved considerably during the immersion times. The ion concentrations released from the samples showed a maximum value during the first day. The concentrations steadily decreased after that. During the first week, the released ion concentrations were higher from the granules than scaffolds, with similar but smaller differences in longer time points. There were no marked differences in the released ion concentrations between Tris and SBF runs.

The cumulative dissolved fraction of silicon is shown in Fig. 4d. The cumulative dissolution curves suggest that more than 40% had dissolved during the 14 days of continuous flow-through immersion.

### In vivo reactions

**Fig. 6:**
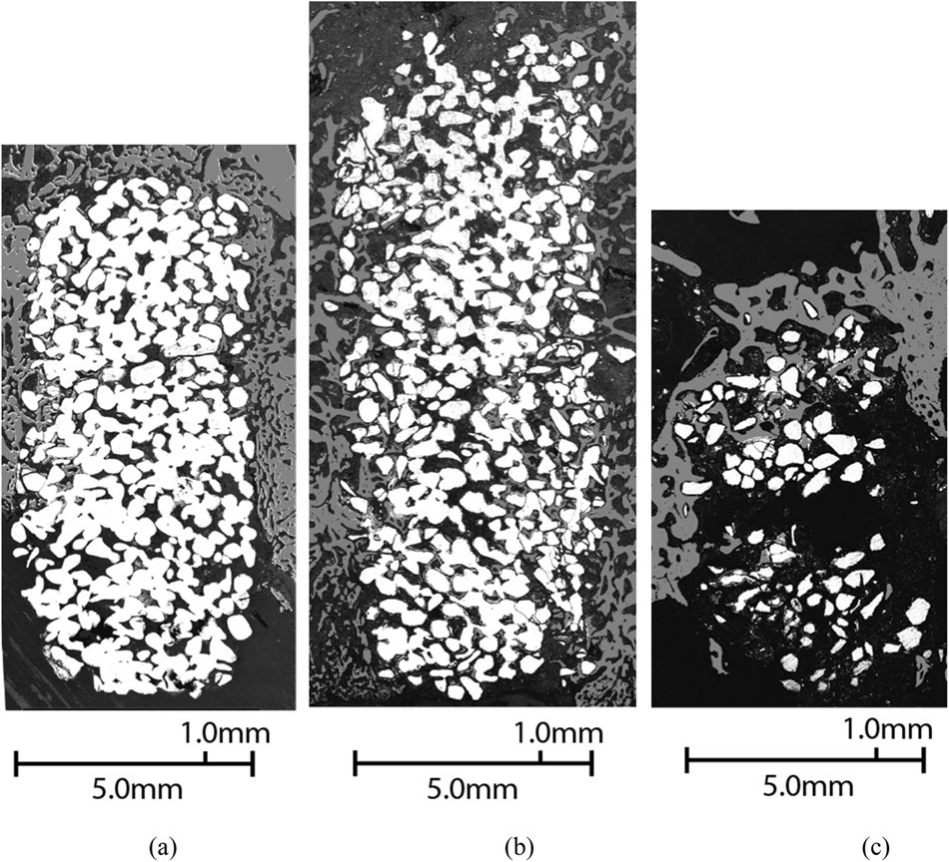
Examples of S59 scaffolds after (**a**) 14 days, (**b**) 28 days, (**c**) 56 days in rabbit femur. The bone around and inside the scaffolds is colored gray; the glass is shown as white

SEM panorama images of the scaffolds in rabbit femurs after 14, 28, and 56 days are shown in Fig. 6a–c. The different reaction layers were identified, and the bone was colored gray to better illustrate bone growth into the scaffolds. The scaffolds showed bone formation mainly around and on the surface of the implants; only minimal reaction layer formation (silica-rich or HA) was identified. The scaffolds were fragile after 56 days of implantation, and only one scaffold could be imaged; the two other scaffolds were too fragile and broke during processing to SEM imaging.

**Fig. 7:**
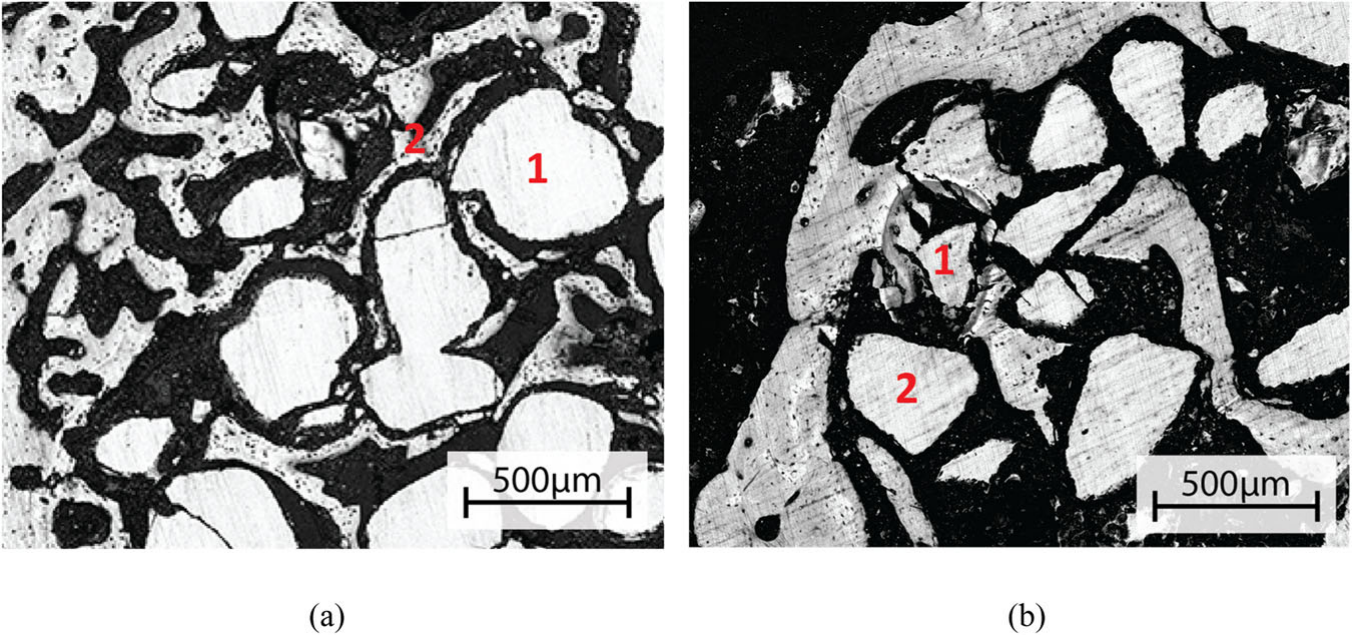
High-magnification SEM images of S59 scaffolds after (**a**) 14 days and (**b**) 56 days in rabbit femur. **a**: Point 1 shows unreacted S59 glass; Point 2 shows new bone formation. **b**: scaffold granule with proper bone attachment in point 1; Point 2 shows a scaffold granule not bonded to surrounding new bone

The higher-magnification SEM image in Fig. 7a shows gaps between bone and implant surface, suggesting that bone had grown into the scaffolds but not bonded to the scaffolds after 14 days. After 56 days of implantation, some parts of the scaffold surface showed typical reaction layers with proper bone attachment (point 1 in Fig. 7b), while no reaction layer but a gap between the glass and the surrounding new bone was identified for most parts of the scaffold (point 2 in Fig. 7b).

## Discussion

The dissolution of sintered glass S59 scaffolds was studied in vitro and in vivo. In vitro, the scaffolds were immersed in SBF and Tris under continuous fluid flow conditions for 14 days, and in vivo, they were implanted in rabbit femurs for up to 56 days.

No notable reaction layers were observed in the SEM images in vitro. Nevertheless, the dissolved ion concentrations showed that the scaffolds dissolved considerably during the immersion. Using the approximations described above, within the 14-day experiment, more than 40% of the silicon initially present in the glasses had dissolved. The dissolution was slightly slower for sintered scaffolds than particles, likely due to the particles’ higher surface area.

This considerable yet congruent dissolution shows the potential of glass S59 to provide beneficial ions to the injury site at a steady rate. As no significant reaction layer formed and no reaction layer would hinder the ion release [51], glass S59 could likely dissolve completely at longer experimental times. As the necks are the thinnest parts, the scaffolds would likely lose their structural integrity at more prolonged exposure in vitro.

The absence of notable reaction layers was also observed in vivo. After 14 and 28 days in the rabbit femur, the scaffolds had partly dissolved, exhibiting bone ingrowth only close to the outer edges of the scaffolds (Fig. 6a, b). The absence of reaction layers was assumed to be due to observed congruent dissolution, providing no nucleation sites for layer precipitation. However, some HA precipitation was observed after 28 days in vivo, implying that the local pH and ion concentrations had increased sufficiently sporadically to allow some HA to precipitate on the scaffold surfaces. Still, as the reaction layer structure enabling bioactive glasses to attach to the new bone tissue requires both silica-rich layers providing HA nucleation sites, the S59 scaffolds were not chemically bonded to the new bone tissue. Therefore, the S59 composition was not bioactive but biocompatible.

After 56 days (Figs. 6c and 7b) the scaffolds had lost their structural integrity due to extensive dissolution and a lack of new bone growth supporting the structure. Therefore, although the scaffolds initially had a compressive strength within the range of cancellous bone (11 MPa, compared to 2–12 MPa for cancellous bone [52]), their strength was drastically reduced during the exposure to body fluids.

The considerable yet stable dissolution for glass S59 differs from what is observed for more established, clinically used bioactive glasses, e.g., 45S5 or S53P4, for which the reactiveness and the reaction layer formation complicate the prediction of long-term dissolution [53]. The advantage of congruent dissolution observed for glass S59 is that the dissolution of therapeutic ions, e.g., Si and Ca species, at the implantation site is steady and predictable. As the scaffolds lost their structure during prolonged in vivo exposure, the composition seemed unsuitable for mono-material load-bearing scaffolds. However, as the scaffolds allowed bone ingrowth and provided stable ion release, the S59 composition could be a candidate for composites, especially if further doped with additional elements, such as Cu, Sr, Zn, or Co, which are known to stimulate cellular processes and provide structural support [10], [38]. Slowly but congruently dissolving glasses could be feasible candidates for other applications, e.g., soft tissue regeneration and wound healing.

The porosity of the scaffolds was low, around 33%. In our previous studies, scaffolds sintered through the same method and granule size provided desired tissue ingrowth with porosities of 50 ± 3% [54] and 49 ± 2% [49]. As the glass S59 dissolved congruently, the scaffolds became increasingly porous throughout the exposure.

Ion dissolution should enhance bone growth into the scaffold to provide additional strength. Here, after 56 days in vivo, the scaffolds were not properly strengthened by bone ingrowth but were too fragile for detailed analyses. Biocompatible glass scaffolds have been reported to lose their strength during static in vitro immersion [55]. In our previous study [56], the compressive strength of S59 scaffolds decreased from the initial value of 11.1 MPa to 9.3 MPa during 4 weeks of static SBF immersion. In the current study, the scaffolds were too fragile for compression testing after the dynamic immersion, implying a more drastic drop in the compressive strength. In static immersion, the total dissolution was much lower, around 5% of total silicon after 28 days [56]. In contrast, the calculated total in vitro silicon dissolution was higher than 40% in the present dynamic study. As HA reaction layer formation was not identified, silicon dissolution was considered to represent the overall glass dissolution.

No qualitative dissolution data could be collected from the in vivo study. The scaffolds had degraded in vivo so considerably that they lost their structure after 56 days. The degradation behavior in vivo was better mimicked with the used set-up, with fresh buffer solution flowing through the sample placed in a reactor cell, than with the static immersion method.

## Conclusions

The dissolution of porous scaffolds of a biocompatible glass S59 was studied in vivo in rabbit femurs and under continuous flow conditions in vitro. Both methods showed that the glass scaffolds dissolved significantly during the 14-day in vitro immersion and 56-day in vivo implantation. The scaffolds allowed bone ingrowth. However, when implanted in rabbit femur metaphyses, the scaffolds did not promote bone ingrowth to replace the strength lost due to congruent glass dissolution. The scaffolds lost their structure and strength as soon as the thinnest parts, the necks, had dissolved. In vitro, S59 dissolved congruently throughout the 14-day experiments. The results suggest that the biocompatible glass S59 could be used in a composite with a slowly biodegrading polymer providing long-term stability. The glass scaffolds can provide initial strength and significant but predictable ion dissolution over longer exposure times. When supplemented with additional appropriate therapeutic ions, this biodegradable glass will likely release a constant dose of the ions to the surrounding solution, which could imply possibilities of using the composition in applications for soft tissue regeneration and wound healing.
